# Genome-Wide Analysis of the Hsf Family and Functional Characterization of *CiHsf10* Under Low-Temperature Stress in *Chrysanthemum indicum*

**DOI:** 10.3390/plants15081149

**Published:** 2026-04-09

**Authors:** Yuzhi Song, Siyu Feng, Xuanlu Liu, Jiayi Yin, Qianru Yu, Lixi Qu, Xue Yang, Yun Bai, Yunwei Zhou

**Affiliations:** College of Horticulture, Jilin Agricultural University, 2888 Xincheng Street, Changchun 130118, China; songyuzhi9@gmail.com (Y.S.); swxfsy66@163.com (S.F.); liuxuanlu0906@163.com (X.L.); yjy15543192507@163.com (J.Y.); 19845479802@163.com (Q.Y.); 18787742541@163.com (L.Q.); yangxue@jlau.edu.cn (X.Y.)

**Keywords:** *Chrysanthemum indicum*, Hsf, *CiHsf10*, low-temperature stress

## Abstract

To improve Chrysanthemum tolerance to low temperatures and its adaptability to low autumn temperatures in Northeast China, we conducted the first genome-wide identification of the heat shock transcription factors (Hsfs) in *Chrysanthemum indicum* under low-temperature stress. Based on genome-wide analyses, we identified 14 *CiHsf* genes in *Chrysanthemum indicum*. Based on structural characteristics, the genes were grouped into two subfamilies, comprising 10 HsfA and four HsfB members, with no representatives of the HsfC subfamily detected. *CiHsf1*~*CiHsf14* were located on seven chromosomes, and their promoter regions harbored numerous cis-acting elements associated with responses to low temperature, hormones, and light. Tissue-specific expression profiling revealed that seven *CiHsf* genes were predominantly expressed in roots, two in stems, three in leaves, and two in flowers. The analysis of low-temperature expression characteristics showed that *CiHsf2*, *CiHsf5*, *CiHsf8*, and *CiHsf10* were significantly upregulated following cold acclimation, indicating that these genes may participate in the low-temperature response mechanism of *Chrysanthemum indicum*. Here, we demonstrated that transient transformation of *Chrysanthemum indicum* with *35S:CiHsf10* reduced reactive oxygen species (ROS) accumulation under low-temperature stress, which may contribute to enhanced cold tolerance.

## 1. Introduction

Plants frequently encounter abiotic stresses, including drought, salinity, high temperatures, and low temperatures during natural growth [1,2]. Among them, low-temperature stress, as a widespread form of stress, can markedly influence plant metabolism, growth, and development, thereby decreasing crop productivity [3,4]. Plants have developed various complex mechanisms to cope with damage caused by low-temperature stress. These mechanisms are largely dependent on tightly controlled gene expression programs, and transcription factors are key regulators of this process.

Among the numerous transcription factor families, heat shock transcription factors (Hsfs) have attracted much attention due to their wide response to various stress conditions [5,6]. In plants, Hsf proteins are relatively conserved and contain multiple structurally and functionally conserved domains, including the N-terminal DNA-binding domain (DBD), the oligomerization domain (HR-A/B), and the C-terminal transcriptional activation motif (AHA), as well as regulatory motifs such as the nuclear localization signal (NLS), nuclear export signal (NES), and repressor domain (RD) [7]. Its N-terminal DBD contains a helix-turn-helix motif that specifically binds to heat shock elements (HSEs) in target gene promoters, leading to activation of stress-responsive gene transcription [8]. According to the length of the linker connecting the DBD and HR-A/B domains and the number of amino acid insertions in the HR-A/B region, plant Hsfs are generally categorized into three classes: HsfA, HsfB, and HsfC [9,10]. The HR-A/B domains of HsfA and HsfC contain 21 and seven additional amino acid residues, respectively, which extend the overall HR-A/B domain structure [6,11]. An AHA motif, essential for transcriptional activation, is present at the C-terminus of HsfA proteins, whereas HsfB and HsfC proteins lack this motif and consequently show no transcriptional activation activity [12]. HsfB members generally act as co-regulators or function as transcriptional repressors [13,14]. Although relatively few studies have focused on HsfC, this subfamily is also involved in stress responses [5].

*Hsf* genes have so far been characterized in the genomes of the majority of plant species, with family size varying across different species. For example, the Hsf gene family has been reported to include 21 members in *Arabidopsis thaliana* [9], 25 in *Oryza sativa* [15], 19 in *Vitis vinifera* [16], 31 in *Zea mays* [17], 25 in *Malus domestica* [18], 52 in *Glycine max* [19], 24 in *Solanum lycopersicum* [20], and 25 in *Capsicum annuum* [21]. Based on this comprehensive identification, functional studies of Hsf gene family members have received considerable attention and have been widely reported. *AtHsfA2* overexpression confers increased tolerance to heat, salt, and osmotic stress in *Arabidopsis thaliana* [22]. Overexpression of *HSFA6s* enhanced heat tolerance in *Triticum aestivum* [23]. A *Populus Hsf* gene was shown to regulate *WRKY1* transcription, leading to enhanced salinity resistance in transgenic *Arabidopsis thaliana* [24]. The roles of Hsfs in cold stress responses have attracted increasing attention. *HbHsfA1* and *HbHsfB1* overexpression markedly improved cold tolerance in *Hevea brasiliensis* [25]. *HSF21* responds to cold stress and promotes low-temperature tolerance in *Zea mays* by maintaining lipid metabolism homeostasis [26]. Heterologous expression of the *CdHsfA9* gene from *Cynodon dactylon* in *Arabidopsis thaliana* enhanced cold tolerance of the transgenic plants [27]. Despite extensive studies of Hsfs in model organisms and important crops, little is known about their functions in ornamental plants.

*Chrysanthemum morifolium* is a globally important flower with both economic and cultural value. As a related species, *Chrysanthemum indicum* is valued not only for its ornamental and medicinal properties but also for its excellent ecological adaptability. In particular, its strong tolerance to abiotic stress, including low temperature, makes it an important germplasm resource for Chrysanthemum resistance breeding. However, the key molecular mechanisms supporting its excellent resistance, particularly highlighting the role of the Hsf gene family in mediating low-temperature stress responses in *Chrysanthemum indicum*, have not been systematically analyzed at the genomic level. Using the whole-genome sequence of *Chrysanthemum indicum*, we conducted a genome-wide identification and characterization of its Hsf gene family [28]. We performed a comprehensive genome-wide analysis of *CiHsf* genes, including analyses of phylogenetic relationships, chromosomal mapping, gene architecture, conserved motifs, promoter cis-acting elements in promoters, and gene synteny. Furthermore, we analyzed their expression patterns in various tissues and in response to low-temperature stress. By uncovering the molecular mechanisms underlying cold tolerance in *Chrysanthemum indicum*, this study offers a theoretical basis and genetic resources for improving cold tolerance in *Chrysanthemum morifolium*, and lays important groundwork for investigating the functions of CiHsfs and elucidating the low-temperature tolerance mechanism in *Chrysanthemum indicum*.

## 2. Results

### 2.1. Genome-Wide Identification and Physicochemical Analysis of CiHsfs

Genome-wide analysis revealed 14 Hsf family members in the *Chrysanthemum indicum* genome through bioinformatics analysis and were designated *CiHsf1* to *CiHsf14* according to their chromosomal positions. A comprehensive analysis of the physicochemical properties was conducted for all proteins, with detailed information summarized in Table S1. The lengths of CiHsf proteins varied from 234 to 496 amino acids, with predicted molecular weights (MW) of 27.37–54.74 kDa. The theoretical isoelectric points (pI) of CiHsf proteins ranged from 4.72 to 9.04, of which 12 members were acidic. Instability indices (II) values of CiHsf proteins varied between 33.00 and 73.30, and only two proteins were predicted to be stable (II < 40). The grand average of hydropathicity (GRAVY) values of the CiHsf proteins varied between −0.847 and −0.359, all of which were negative, and their negative values indicate that all members are hydrophilic. CiHsf proteins exhibited aliphatic index values ranging from 65.80 to 82.48. All 14 CiHsf proteins were predicted to be localized in the nucleus according to the Plant-mPLoc server, indicating that CiHsf proteins mainly exert their functions in the nucleus. Functional domains of CiHsf proteins, including the DBD, HR-A/B region, NLS, NES, and AHA motifs, are summarized in Table S2.

### 2.2. Phylogenetic Relationships of CiHsf Gene Family

**Figure 1:**
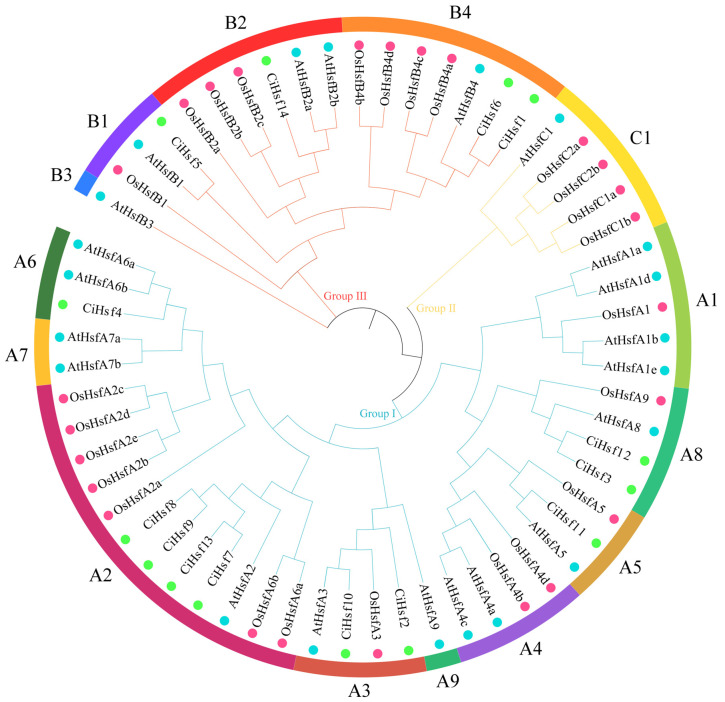
Phylogenetic analysis of the Hsf family among *Chrysanthemum indicum* (Ci), *Arabidopsis thaliana* (At), and *Oryza sativa* (Os). The Hsf proteins are clustered into three main groups (Group I, II, and III) and further divided into subfamilies (A1–A9, B1–B4, and C1), indicated by the colored arcs on the outer circle. The colored solid circles at the branch tips represent different species: green for *Chrysanthemum indicum*, blue for *Arabidopsis thaliana*, and pink for *Oryza sativa*. Full-length Hsf protein sequences from the three species are provided in Table S5.

To investigate the evolutionary relationships and potential functional divergence of the Hsf gene family in *Chrysanthemum indicum*, Hsf protein sequences from *Chrysanthemum indicum* (14), *Arabidopsis thaliana* (21), and *Oryza sativa* (26) were used to construct a phylogenetic tree (Figure 1). Based on phylogenetic analysis, the Hsf proteins were classified into three primary groups, referred to as Groups I, II, and III, indicating that the Hsf family has undergone functional divergence throughout its evolutionary history. According to the established classification system of *Arabidopsis thaliana* and *Oryza sativa*, the three major groups were further divided into three subfamilies: Group I corresponded to the HsfA subfamily, Group II to the HsfC subfamily, and Group III to the HsfB subfamily. Within the *CiHsf* gene family, the HsfA subfamily represented the largest group, consisting of 10 genes. Among these, *CiHsf7*, *CiHsf8*, *CiHsf9*, and *CiHsf13* clustered closely together, suggesting that they share conserved evolutionary lineages and may possess functional homology. The HsfB subfamily comprised 4 *CiHsf* members. Notably, no members of the HsfC subfamily were identified in *Chrysanthemum indicum*, indicating the loss of this subfamily during evolution. Based on the phylogenetic tree topology, *CiHsf* genes clustered with AtHsf and OsHsf genes within the same branches, implying that these genes may share conserved functions with their orthologs in *Arabidopsis thaliana* and *Oryza sativa*.

### 2.3. Chromosomal Locations of CiHsf Genes

**Figure 2:**
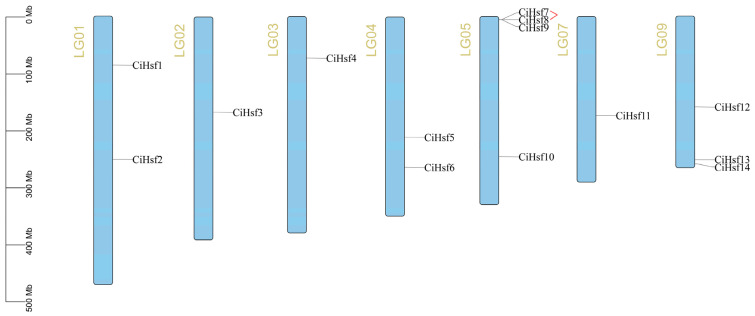
Chromosomal distribution of 14 *CiHsf* genes in *Chrysanthemum indicum*. Each chromosome is labeled with its name on the left. Red lines indicate tandem duplication between *CiHsf* gene pairs.

According to the *Chrysanthemum indicum* genome annotation, the 14 *CiHsf* genes were localized on seven chromosomes, exhibiting a non-uniform distribution (Figure 2). The largest number of *CiHsf* genes (4) was located on chromosome 5, including a pair of tandemly duplicated genes (*CiHsf7* and *CiHsf8*). Chromosome 9 contained 3 *CiHsf* genes, while chromosomes 1 and 4 each carried 2. Chromosomes 2, 3, and 7 each harbored 1 *CiHsf* gene.

### 2.4. Analysis of Gene Structure and Conserved Motifs in CiHsf Genes

**Figure 3:**
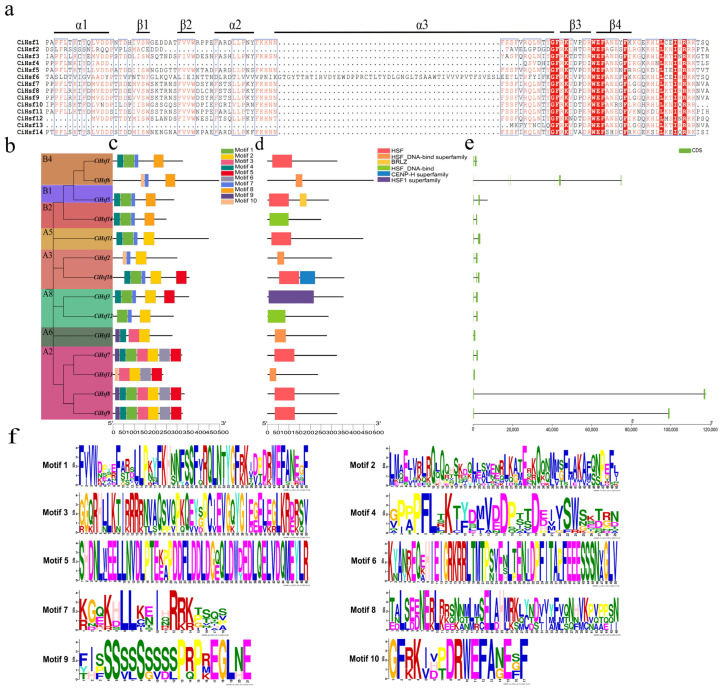
Analyses of *CiHsf* genes, including multiple sequence alignment, phylogenetic relationships, conserved motifs, protein domains, and gene structures. (**a**) Multiple sequence alignment of the DBDs of the CiHsf protein family. (**b**) Phylogenetic tree of 14 CiHsf proteins. (**c**) Motif compositions of the CiHsf proteins. Ten conserved motifs are displayed using boxes of different colors. (**d**) Conserved domain analysis of the CiHsf proteins. Protein length can be estimated by the bottom scale. (**e**) Gene structure analysis. Exons (coding sequences, CDS) are represented by green boxes, whereas introns are indicated by black lines. (**f**) Conservative motif logo.

Sequence conservation among *CiHsf* genes was examined by analyzing conserved motifs across all family members. The amino acid sequences of the 14 identified CiHsf proteins were subjected to multiple sequence alignment. Analysis revealed that the highly conserved DBD of CiHsf proteins, about 100 residues long, contained 3 α-helices and 4 β-sheets organized as α1–β1–β2–α2–α3–β3–β4 (Figure 3a). Such structural characteristics facilitate the precise recognition and binding of HSEs.

To explore the evolutionary relationships of the 14 *CiHsf* genes, phylogenetic analysis was performed in conjunction with examinations of the phylogenetic tree (Figure 3b). The 14 *CiHsf* genes were classified into 8 subgroups, consistent with the evolutionary relationship analysis described in Section 2.2. Among these subgroups, A2 represented the largest clade, containing 4 *CiHsf* genes, while the remaining subgroups each comprised 1 or 2 *CiHsf* genes.

To further characterize the protein structure of CiHsf family members, using the MEME Suite, 10 conserved motifs (motifs 1–10) were identified across the 14 CiHsf proteins (Figure 3c). Each CiHsf protein contained between 3 and 7 conserved motifs, with motifs 1, 2, and 4 present in most members, indicating that these three motifs are evolutionarily conserved. As expected, proteins with closer phylogenetic relationships exhibited similar motif compositions. Analysis of conserved domains showed that all CiHsf proteins harbor a canonical HSF-type DBD (Figure 3d), confirming their identity as members of the Hsf family.

Exon-intron structure analysis revealed significant structural variation among *CiHsf* genes (Figure 3e). *CiHsf* genes contained between 1 and 8 exons, accompanied by 1 to 7 introns. Among them, *CiHsf6* possessed the most complex structure, containing 8 exons and 7 introns. *CiHsf13* displayed the simplest structure with only 1 exon. Most of the *CiHsf* genes contained 2 exons, and notable differences in exon positions and structures were observed across different subgroups, reflecting considerable structural diversity. Collectively, these analyses demonstrated that *CiHsf1* through *CiHsf14* possess distinct structural patterns, while Consistent exon–intron architectures and motif compositions were observed among members of the similar phylogenetic subgroup.

### 2.5. Cis-Acting Element Analysis of CiHsf Gene Promoters

**Figure 4:**
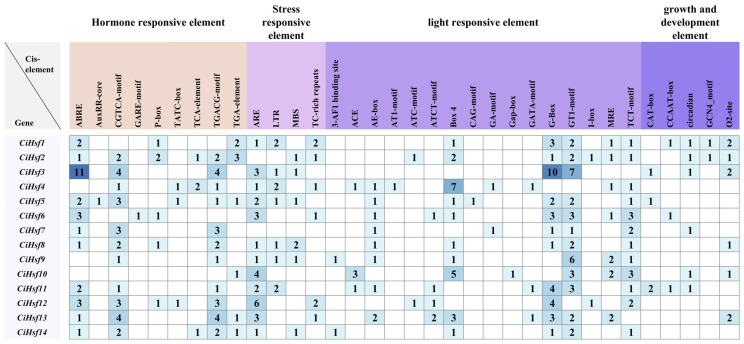
Distribution of cis-acting elements in the promoters of *CiHsf* genes. The numbers within the squares and the color intensity indicate the quantity of cis-acting elements. Abbreviation definitions and functions: Hormone-responsive: ABRE (abscisic acid response), AuxRR-core/TCA-element (salicylic acid response), CGTCA-motif/TGACG-motif (meJA response), GARE-motif/p-box/TATC-box (gibberellin response), TGA-element (Auxin response). Stress-responsive: ARE (anaerobic induction), LTR (low-temperature response), MBS (MYB binding site involved in drought-inducibility), TC-rich repeats (defense and stress response). Light-responsive: 3-AF1 binding site, ACE, AE-box, AT1-motif, ATC-motif, ATCT-motif, Box 4, CAG-motif, GA-motif, Gap-box, GATA-motif, G-box, GT1-motif, I-box, MRE, TCT-motif. Growth and Development: CAT-box (meristem expression), CCAAT-box (MYBHv1 binding site), circadian (circadian control), GCN4-motif (endosperm expression), O2-site (zein metabolism regulation).

The 2000 bp upstream sequences of the 14 *CiHsf* genes were analyzed, resulting in the identification of 34 cis-acting regulatory elements. The identified cis-acting elements were functionally classified into 4 groups: hormone-responsive, stress-responsive, light-responsive, and growth and development-related elements (Figure 4). *CiHsf* genes harbored varying numbers of cis-acting elements, ranging from 9 to 18 per gene. All 14 *CiHsf* genes contained light-responsive elements. Among them, 12 genes harbored anaerobic response elements (ARE), 11 contained abscisic acid (ABA) and methyl jasmonate (MeJA) response elements, 7 possessed low-temperature response elements (LTR), and 6 included auxin response elements as well as defense and TC-rich repeats related to defense and stress. The results suggest that *CiHsf* genes may play important regulatory roles in hormone signaling, stress adaptation, light response, and growth and developmental processes in *Chrysanthemum indicum*.

### 2.6. Evolutionary Analysis of CiHsf Genes

**Figure 5:**
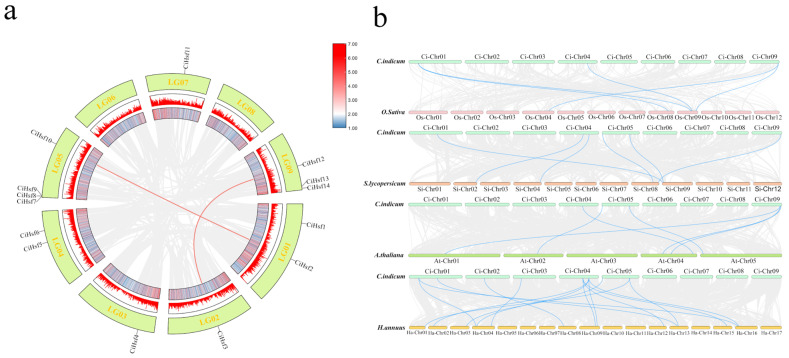
(**a**) Interchromosomal relationships of *CiHsf* genes in *Chrysanthemum indicum*. Red lines indicate the 2 putative segmental duplication gene pairs. (**b**) Comparative tandem duplication analysis of Hsf genes between *Chrysanthemum indicum* and 4 representative plant species. Chromosomes of the four representative plant species are depicted as rectangles in different colors. Gray lines indicate genome-wide collinearity between *Chrysanthemum indicum* and 4 representative plant species, whereas blue lines mark the tandemly duplicated Hsf gene pairs.

To analyze the phylogenetic relationships of *CiHsf* genes, syntenic analysis was performed to identify duplication events within the *Chrysanthemum indicum* genome (Figure 5a). Analysis of gene duplication revealed two segmentally duplicated gene pairs—*CiHsf2*/*CiHsf10* and *CiHsf3*/*CiHsf12*—distributed across four chromosomes. These findings suggest that these 2 gene pairs originated from segmental duplication events and may have evolved from common ancestral genes.

To investigate the evolutionary conservation of *CiHsf* genes, syntenic relationships were analyzed between *Chrysanthemum indicum* and four representative species: *Arabidopsis thaliana*, *Oryza sativa*, *Solanum lycopersicum*, and *Helianthus annuus* (Figure 5b). Synteny analysis indicated that 5 *CiHsf* genes are conserved in syntenic blocks with Hsf genes in *Arabidopsis thaliana*, 3 with those in *Oryza sativa*, 6 with *Solanum lycopersicum*, and 8 with *Helianthus annuus*. Remarkably, the largest number of syntenic gene pairs occurred between *Chrysanthemum indicum* and *Helianthus annuus*, both members of the Asteraceae family, reflecting their closer phylogenetic relationship. In contrast, the fewest syntenic pairs were detected between *Chrysanthemum indicum* and the monocotyledonous species *Oryza sativa*, indicating that the conservation and number of Hsf homologs correspond to the evolutionary distances among species, with more closely related species exhibiting higher synteny.

### 2.7. Expression Pattern Analysis of CiHsf Genes in Different Tissues

**Figure 6:**
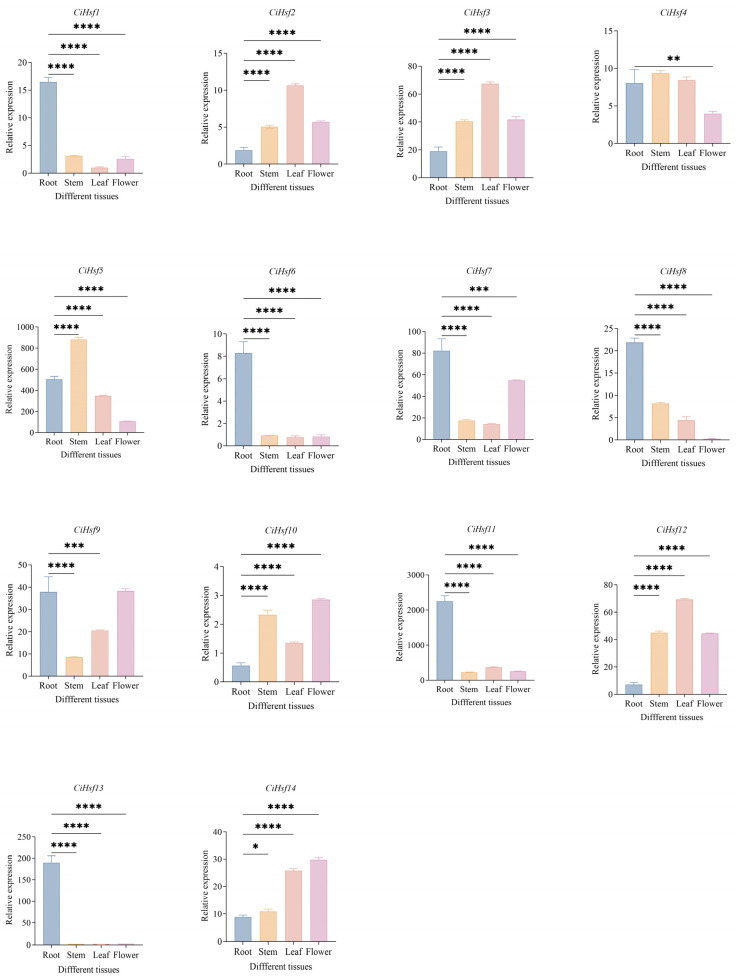
Tissue-specific expression patterns of CiHsfs. Gene names are displayed above each histogram, and the associated tissues are labeled below. Error bars indicate SD from three independent replicates. * *p* < 0.05, ** *p* < 0.01, *** *p* < 0.001, **** *p* < 0.0001.

As a multi-member gene family, different members of *CiHsf* genes often achieve functional diversification and fine regulation through tissue-specific and spatiotemporal expression patterns. To investigate the potential functional differences in the *CiHsf* gene family during the growth and development of *Chrysanthemum indicum*, for qRT-PCR analysis, independent biological replicates were collected from 4 tissues of *Chrysanthemum indicum*: roots, stems, leaves, and flowers (Figure 6). The expression patterns of *CiHsf* genes varied considerably among different tissues. Among them, *CiHsf1*, *CiHsf6*, *CiHsf7*, *CiHsf8*, *CiHsf9*, *CiHsf11* and *CiHsf13* were mainly expressed in roots, while *CiHsf5* displayed strong expression in flowers. *CiHsf2*, *CiHsf3*, *CiHsf10*, *CiHsf12*, and *CiHsf14* showed elevated expression in stems, leaves, and flowers. The above shows that the *CiHsf* genes show diverse and complex expression profiles across different tissues of *Chrysanthemum indicum*.

### 2.8. Expression Pattern Analysis of CiHsf Genes Under Low-Temperature Stress

**Figure 7:**
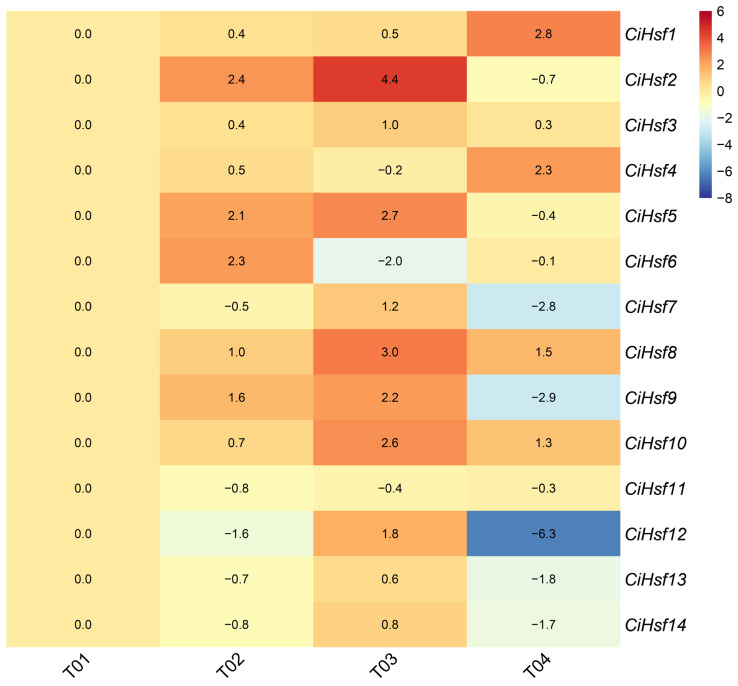
Expression patterns of *CiHsf* genes in *Chrysanthemum indicum* under low-temperature stress. T01, without low-temperature treatment. T02, direct transfer to −4 °C for 4 h. T03, 4 °C for 4 h, followed immediately by −4 °C for 4 h. T04, 4 °C for 4 h. Gene expression levels are indicated by a color gradient from blue (low) to red (high). The values in the cells represent the log_2_ fold changes in other treatment groups relative to the T01 treatment group.

To elucidate the response mechanisms of *CiHsf* genes to low-temperature stress, their expression patterns were analyzed under different cold treatment conditions. The RNA-Seq analysis identified that *CiHsf* genes respond differentially to cold stress (Figure 7). The results revealed that several genes exhibited distinct expression specificities. Notably, *CiHsf2*, *CiHsf5*, *CiHsf8*, and *CiHsf10* exhibited significant up-regulation in response to the T03 treatment, with them exhibiting 4.4-fold, 2.7-fold, 3.0-fold, and 2.6-fold increases in expression compared with the T01 control, respectively. These results suggest that these genes may contribute to the response of *Chrysanthemum indicum* to low-temperature stress. Overall, the T03 treatment induced widespread up-regulation of gene expression, with mean expression levels significantly exceeding those observed in the other treatments, indicating that the Hsf signaling pathway was likely activated under this cold acclimation condition.

### 2.9. CiHsf10 May Play a Role in the Response to Low-Temperature Stress

**Figure 8:**
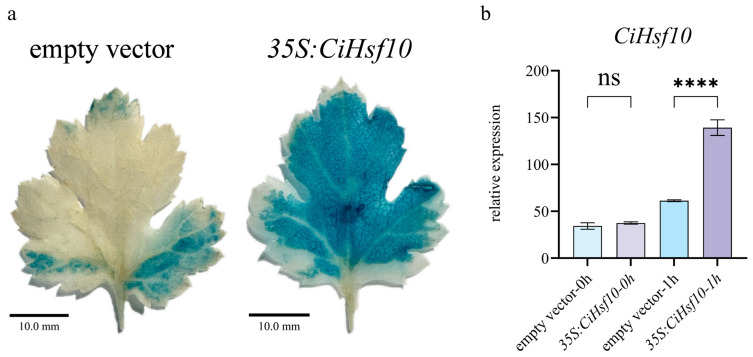
Analysis of *35S:CiHsf10* transiently transformed plants by GUS staining and qRT-PCR. (**a**) Representative images showing GUS staining in plants transiently transformed with the empty vector or *35S:CiHsf10*. Scale bar = 10.0 mm. (**b**) *CiHsf10* expression in plants transiently transformed with the empty vector or *35S:CiHsf10*. The labels 0 h and 1 h indicate plants exposed to 4 °C for 0 and 1 h, respectively. **** *p* < 0.0001, ns, not significant.

Because HsfA plays an important role in cold stress response [27], we hypothesized that *CiHsf10* might function under low-temperature stress. To construct the *35S:CiHsf10* overexpression construct, the coding sequence (CDS) of *CiHsf10* was amplified and cloned into the pBI121 vector. Transient transformation of *Chrysanthemum indicum* plants was performed using either the empty pBI121 vector (control) or the *35S:CiHsf10* construct. Successful transient transformation was verified using GUS staining and qRT-PCR analysis of *CiHsf10* expression. GUS staining was performed on leaves of *Chrysanthemum indicum* transiently transformed with either the empty vector (pBI121) or the *35S:CiHsf10* construct. The staining intensity was stronger in leaves overexpressing *CiHsf10* compared to the empty vector control (Figure 8a). At 72 h post-transformation, plants grown under normal conditions were treated with low temperature treatment for 0 and 1 h, and *CiHsf10* expression levels were measured by qRT-PCR. Under normal conditions (0 h), *CiHsf10* expression did not differ significantly between *35S:CiHsf10*-overexpressing and empty vector-transformed plants. Following 1 h of low-temperature treatment, *CiHsf10* expression was markedly higher in the *35S:CiHsf10*-overexpressing plants compared with empty vector controls (Figure 8b).

**Figure 9:**
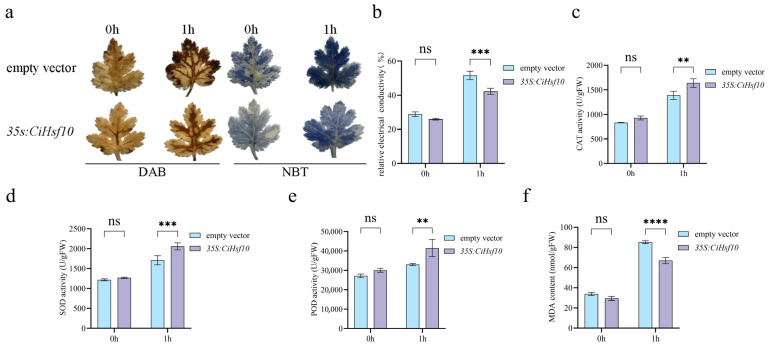
*35S:CiHsf10* transiently transformed plants have fewer ROS products in *chrysanthemum*. (**a**) DAB and NBT histochemical staining. (**b**) Relative electrolyte leakage under low-temperature stress; (**c**) CAT activity under low-temperature stress. (**d**) SOD activity under low-temperature stress. (**e**) POD activity under low-temperature stress. (**f**) Analysis of MDA under low-temperature stress. ** *p* < 0.01, *** *p* < 0.001, **** *p* < 0.0001, ns, not significant.

Diaminobenzidine (DAB) and nitroblue tetrazolium (NBT) histochemical staining were performed to detect ROS accumulation in leaves of *Chrysanthemum indicum* transiently transformed with empty vector (pBI121) and the *35S:CiHsf10* construct (Figure 9a). Under low-temperature stress, staining spots increased, and leaf color deepened, indicating elevated ROS accumulation. Notably, leaves overexpressing *CiHsf10* exhibited fewer brown (DAB) and blue (NBT) spots and showed reduced oxidative damage compared to empty vector controls. To further evaluate the physiological effects of *CiHsf10* overexpression under low-temperature stress, relative electrical conductivity (REC), catalase (CAT) activity, superoxide dismutase (SOD) activity, peroxidase (POD) activity, and malondialdehyde (MDA) content were measured in *Chrysanthemum indicum* leaves transiently transformed with empty vector (pBI121) or the *35S:CiHsf10* construct. Under low-temperature stress, all measured parameters were elevated compared to normal conditions in both groups. Significantly elevated activities of CAT, SOD, and POD were observed in *35S:CiHsf10*-overexpressing plants relative to empty vector-transformed controls (Figure 9c–e). Furthermore, after low temperature treatment, significantly reduced REC and MDA contents were observed in *35S:CiHsf10*-overexpressing *Chrysanthemum indicum* leaves compared with empty vector-transformed controls (Figure 9b,f). *CiHsf10* appears to reduce oxidative damage caused by low-temperature stress by promoting the activity of the antioxidant system, suggesting a role in enhancing the cold tolerance of transgenic plants.

## 3. Discussion

Hsf genes are widespread in plants and have been reported to play essential roles in growth, development, and responses to various stresses. They primarily contribute to abiotic stress tolerance through the signal transduction pathway [29], Copolymer pathway [30], protein phosphorylation [31] and ubiquitination pathway [32]. Following the release of the complete genome sequence of *Chrysanthemum indicum*, we performed the first genome-wide identification and characterization of its Hsf gene family in this species, leading to the identification of 14 *CiHsf* genes. Compared with *Arabidopsis thaliana* (21) [9] and *Zea mays* (31) [17], there are differences in the number. However, as a terrestrial plant, *Chrysanthemum indicum* possesses a greater number of Hsf genes than algae, which is consistent with the reported evolutionary trend [33,34,35]. Previous studies indicate that differences in Hsf gene numbers across species are linked to whole-genome duplication (WGD). During its evolutionary history, *Chrysanthemum indicum* is thought to have experienced up to two WGD events; by comparison, *Arabidopsis thaliana* and *Zea mays* have each undergone three or more WGD events [36,37]. It is generally accepted that the diversity of Hsf genes in plants may result from gene duplication and WGD events at different evolutionary stages, followed by extensive gene loss [6]. The *CiHsf* gene family may therefore represent a product of adaptive evolution, potentially contributing positively to the enhancement of plant stress tolerance.

Evaluation of the physicochemical characteristics of the CiHsf proteins showed that all 14 members of this gene family are hydrophilic, with 12 predicted to be unstable—a finding consistent with most previous studies [38,39]. Biologically, unstable proteins are likely to be more readily detected and regulated by intracellular regulatory systems. Upon exposure to environmental stress, this instability may facilitate the rapid adjustment of Hsf protein levels within cells. The pI of CiHsf proteins range from 4.72 to 9.04, which closely aligns with the theoretical pI values reported for Hsf family members in most studies [40]. CiHsf proteins are predominantly localized to the nucleus, consistent with their function as transcription factors regulating gene expression in this compartment. Structural differences among gene family members provide key insights into their functional divergence. Analysis of protein motifs revealed that motif 2 is specific to the CiHsfA subfamily, while motif 8 is specific to the CiHsfB subfamily, indicating that the two subfamilies may be involved in distinct functional regulatory pathways.

Phylogenetic trees are essential tools for revealing evolutionary relationships among genes or species. Based on the classification system established in *Arabidopsis thaliana*. In *Verbena bonariensis* and *Cerasus humilis*, the Hsf gene families are divided into three subfamilies (A, B, and C), encompassing 14 distinct classes [41,42]. In contrast, the *CiHsf* gene family identified in this study notably lacks the HsfC subfamily, as well as the A1, A4, A7, A9, and B3 subgroups (Figure 1). This may reflect the limited expansion of the *CiHsf* gene family, suggesting that gene loss occurred during evolutionary processes [43]. The 14 *CiHsf* genes share high homology with those of *Arabidopsis thaliana*, which suggests that the *CiHsf* gene family is conserved through evolution and exhibits close sequence similarity with related species.

The evolution and functional diversification of gene families are largely driven by gene duplication, providing raw genetic material for the emergence of new functions through mechanisms such as tandem duplication, segmental duplication, and whole-genome duplication [44,45]. In the *Chrysanthemum indicum* genome, one tandem duplication event and two segmental duplication events were detected, implying that gene duplication has contributed substantially to the growth and evolutionary development of the *CiHsf* gene family. Comparative analysis of syntenic relationships between *Chrysanthemum indicum* and representative monocot, dicot, model, and Asteraceae plants revealed three, six, five, and eight collinear gene pairs with *Oryza sativa*, *Solanum lycopersicum*, *Arabidopsis thaliana*, and *Helianthus annuus*, respectively. The comparison between *Chrysanthemum indicum* and *Oryza sativa* revealed the smallest number of collinear gene pairs, indicating that the genomes of monocotyledonous and dicotyledonous plants have undergone extensive rearrangement, duplication, and loss events since their divergence approximately 200 million years ago, resulting in a marked reduction in homologous gene blocks. This finding is consistent with previous studies [46,47]. *Chrysanthemum indicum* exhibits the most collinear gene pairs with *Helianthus annuus*, a fellow member of the Asteraceae family. This finding provides direct evidence for the general principle that closely related species generally exhibit higher levels of conservation in genome structure and gene order [48].

Hsf proteins regulate multiple plant developmental processes and integrate various environmental stress signals. Cis-acting element analysis of the *CiHsf* gene promoters revealed the presence of multiple abiotic stress-responsive elements, including cis-elements associated with low-temperature stress (LTR), drought response (MBS), and hormone signaling pathways, such as methyl jasmonate (CGTCA-motif), abscisic acid (ABRE), and salicylic acid (TCA-element). Collectively, the results indicate that *CiHsf* genes may function in stress responses mediated by hormonal signaling. Under stress conditions, plants often accumulate excessive ROS, which can cause oxidative damage to cells. Methyl jasmonate (MeJA) can activate the antioxidant system in plants, thereby improving the efficiency of ROS scavenging [49]. Expression analysis under low-temperature stress revealed differential expression of individual *CiHsf* members in response to cold treatment. Among them, the T03 treatment induced the most extensive up-regulation of gene expression, with *CiHsf2*, *CiHsf5*, *CiHsf8*, and *CiHsf10* showing particularly marked increases, reaching 4.4-fold, 2.7-fold, 3.0-fold, and 2.6-fold of their expression levels at T01, respectively. The pronounced responses observed under the T03 treatment further support its suitability as a key condition for investigating cold stress-related gene function. These findings indicate that cold acclimation may enhance tolerance to subsequent low-temperature stress by triggering the Hsf signaling pathway, which is consistent with the established molecular mechanism of plant cold acclimation.

Tissue-specific gene expression enables plants to respond to abiotic stresses while controlling growth and developmental processes [50,51]. In *Verbena bonariensis* and *Medicago sativa*, Hsf genes have been shown to exhibit differential expression [41,52]. qRT-PCR analysis revealed that high expression of seven *CiHsf* genes (*CiHsf1*, *CiHsf6*, *CiHsf7*, *CiHsf8*, *CiHsf9*, *CiHsf11*, and *CiHsf13*) in roots, suggesting their potential involvement in root-specific environmental adaptation or developmental regulation. The elevated expression of *CiHsf5* in flowers suggests that it may contribute to stress tolerance during floral organ development or reproductive processes. Additionally, *CiHsf2*, *CiHsf3*, *CiHsf10*, *CiHsf12*, and *CiHsf14* expression was relatively high in stems, leaves, and flowers, implying widespread expression among different tissues. These genes may participate in basal physiological metabolism in photosynthetic tissues or mediate responses induced by environmental stresses such as strong light and low temperature affecting aboveground organs. Notably, *CiHsf2* and *CiHsf10* were also significantly induced under low-temperature stress, further supporting their potential functions in cold response in aboveground tissues. Collectively, our results indicate that members of the *CiHsf* gene family are involved in multiple aspects of growth, development, and environmental adaptation of *Chrysanthemum indicum*.

The HsfA3 subgroup is widely recognized as a major regulator of the heat stress response, contributing to protein homeostasis through the activation of heat shock proteins and ROS-scavenging enzyme–encoding genes [53,54]. Low-temperature stress is known to induce oxidative damage; we speculate that the HsfA3 subgroup may additionally participate in cold stress responses. *CiHsf10*, identified in this study, belongs to the HsfA3 subgroup. The selection of *CiHsf10* was not solely based on induction magnitude, but also considered phylogenetic classification, expression stability, and experimental feasibility. Physiological analysis of *35S:CiHsf10* transfected of *Chrysanthemum indicum* at low temperature is detected. The transient expression of this gene significantly reduces ROS accumulation under low-temperature stress, suggesting that HsfA3 may participate in non-heat stress response pathways.

## 4. Materials and Methods

### 4.1. Plant Materials and Exposure to Low-Temperature Stress

*Chrysanthemum indicum* L. (IPNI LSID: 193510-1) plants were grown at the Ornamental Horticultural Germplasm Resource Garden, College of Horticulture, Jilin Agricultural University. For tissue-specific expression profiling, roots, stems, leaves, and flowers were harvested at the flowering stage, frozen in liquid nitrogen, and stored at −80 °C until further analysis. For low-temperature stress treatment, 7~8-leaf-stage seedlings were grown under controlled conditions (25 °C, 16 h light/8 h dark photoperiod) and subjected to the following treatments: T01 (control, untreated); T02 (direct transfer to −4 °C for 4 h); T03 (4 °C for 4 h, followed immediately by −4 °C for 4 h); and T04 (4 °C for 4 h). The temperature gradient (T01–T04) was designed to simulate progressive chilling to freezing stress conditions. In particular, the T03 treatment represents a critical transition from chilling to freezing stress and was selected as a representative condition due to its strong induction of physiological and transcriptional responses.

### 4.2. Genome-Wide Analysis and Identification of Hsf Genes in Chrysanthemum Indicum

Genome and GFF annotation data for *Chrysanthemum indicum* were downloaded from the Chrysanthemum genome database (https://cgd.njau.edu.cn/asteraceae/blast/blastPage, 27 December 2025) [55]. Two complementary strategies were employed to identify Hsf gene family members. First, Hsf protein sequences from *Arabidopsis thaliana* were retrieved from the TAIR database (https://www.arabidopsis.org/, 27 December 2025) and employed as queries for BLASTp searches against the *Chrysanthemum indicum* genome by BLAST (v2.16.0). Second, HMMER (v3.0) was used to perform hidden Markov model (HMM) profiling based on the Hsf domain (Pfam: PF00447) [56] to search for candidate CiHsf proteins. Redundant sequences were removed, and the remaining candidates were subjected to conserved domain validation using the CDD (https://www.ncbi.nlm.nih.gov/Structure/cdd/wrpsb.cgi, 28 December 2025) and SMART (https://smart.embl.de/, 28 December 2025). Sequences without a completely conserved Hsf domain were discarded from further analysis.

### 4.3. Analysis of Physicochemical Properties and Subcellular Localization of CiHsf Proteins

The ExPASy ProtParam tool (https://www.expasy.org/resources/protparam, 29 December 2025) was used to determine the physicochemical properties of CiHsf proteins, including molecular weight, theoretical pI, instability index, and GRAVY [57]. The subcellular localization of the CiHsf proteins was predicted using the Plant-mPLoc server (http://www.csbio.sjtu.edu.cn/bioinf/plant-multi/, 29 December 2025) [58].

### 4.4. Phylogeny, Conserved Motifs, and Gene Structure Analysis

Phylogenetic analysis was conducted to assess the evolutionary relationships among Hsf proteins from *Chrysanthemum indicum*, *Arabidopsis thaliana*, and *Oryza sativa*. The Hsf protein sequences of *Arabidopsis thaliana* and *Oryza sativa* were obtained from TAIR (https://www.arabidopsis.org/, 29 December 2025) and UniProt (https://www.uniprot.org/, 29 December 2025), respectively. ClustalW in MEGA X (v10.1.8) was used to perform multiple sequence alignments [59]. Maximum Likelihood (ML) phylogenetic analysis with 1000 bootstrap replicates was performed, and the resulting tree was visualized and annotated using the iTOL server (https://itol.embl.de/, 30 December 2025). Conserved domain analysis of the CiHsf protein sequences was performed using the NCBI Batch CD-Search web server (https://www.ncbi.nlm.nih.gov/Structure/bwrpsb/bwrpsb.cgi, 2 January 2026). Conserved motifs in CiHsf proteins were identified using the MEME Suite (https://meme-suite.org/meme/, 2 January 2026) [60], setting the maximum number of motifs to 10. Exon–intron organization was inferred from the *Chrysanthemum indicum* GFF annotation file containing genomic DNA and coding sequence (CDS) information. TBtools was used to integrate and visualize all results [61].

### 4.5. Chromosomal Localization of CiHsf Genes

The chromosomal locations of the 14 identified *CiHsf* genes were determined using the *Chrysanthemum indicum* genome annotation files (GFF format). The *CiHsf* genes were named sequentially according to their order on chromosomes, oriented from the short to the long arm. A chromosomal distribution map was generated using TBtools (v2.458) software. In addition, tandem duplication of *CiHsf* genes was recognized based on genomic position criteria.

### 4.6. Identification and Analysis of Cis-Acting Elements in CiHsf Promoters

To investigate the transcriptional regulatory mechanisms underlying the *CiHsf* genes, for each *CiHsf* gene, a 2000 bp sequence upstream of the translation start site (ATG) was extracted from the *Chrysanthemum indicum* genome and considered the putative promoter region. The extracted promoter sequences were analyzed using the PlantCARE database (https://bioinformatics.psb.ugent.be/webtools/plantcare/html/, 3 January 2026) to predict cis-acting regulatory elements. The predicted elements were subsequently classified according to their potential functions, with emphasis on those involved in stress responsiveness, hormone signaling, and developmental processes.

### 4.7. Collinearity Analysis of CiHsf Genes

To examine the syntenic relationships of *Hsf* genes between *Chrysanthemum indicum* and representative plant species, including model plants, monocotyledons, dicotyledons, and Asteraceae species, cross-species collinearity analysis was performed. The *Arabidopsis thaliana* genome sequence and corresponding annotation files were downloaded from TAIR (https://www.arabidopsis.org/, 29 December 2025), while those for *Oryza sativa*, *Solanum lycopersicum*, and *Helianthus annuus* were acquired from UniProt (https://www.uniprot.org/, 29 December 2025). Inter-species synteny analysis of Hsf genes was conducted using the “One Step MCScanX-Super Fast” plugin in TBtools software, and syntenic relationships were visualized using the built-in homology visualization tool in TBtools.

### 4.8. Total RNA Extraction and Quantitative Real-Time PCR (qRT-PCR) Analysis

Total RNA was extracted from roots, stems, leaves, and flowers of *Chrysanthemum indicum* using the TransZol Up Plus RNA Kit (TransGen Biotech, Beijing, China). Library construction and sequencing were performed by Novogene (Beijing, China) on the Illumina platform. For data analysis, raw reads were filtered to remove adapters and low-quality sequences using Trimmomatic. The resulting clean reads were mapped to the *Chrysanthemum indicum* reference genome using HISAT2. Gene expression levels were calculated as FPKM (Fragments Per Kilobase of transcript per Million mapped reads). Differentially expressed genes (DEGs) were identified using the DESeq2 R package, with an adjusted *p*-value < 0.05 and |log_2_FoldChange| ≥ 1 as the thresholds. RNA quality and concentration were assessed using 1% agarose gel electrophoresis and an ultra-micro spectrophotometer (Implen GmbH, Munich, Germany). cDNA was synthesized from qualified RNA using the NovoScript^®^ Plus All-in-one 1st Strand cDNA Synthesis SuperMix (Novoprotein, Suzhou, China), quantified at 500 ng, and stored at −20 °C. Fluorescent quantitative primers were designed using Primer Premier 5.0 (Table S3). The *EF1α* (GenBank accession No. KF305681) served as the internal reference gene [62]. To normalize the internal variation among samples, the *EF1α* gene was used as the internal reference (normalization factor), as it has been validated for stable expression in *C. indicum* under various conditions. Quantitative real-time PCR was performed using a qTOWER 3G real-time PCR system (Analytik Jena, Jena, Germany). The 2^−ΔΔCT^ method was used to calculate the relative expression of target genes. Each experiment was carried out in three biological replicates.

### 4.9. Transient Transformation of CiHsf10 and Plant Analysis of Transient Transformation Under Low-Temperature Stress

The full-length coding sequence of *CiHsf10* was amplified from *Chrysanthemum indicum* cDNA using gene-specific primers designed in Primer Premier 5.0 (Table S4). The overexpression construct *35S:CiHsf10* was generated using the pBI121 vector, and GUS staining was performed to validate successful transient transformation. For transient transformation, *Chrysanthemum indicum* seedlings were submerged in an infiltration medium containing *Agrobacterium tumefaciens* (OD600 = 0.6, pH 5.6) and subjected to vacuum infiltration (8 kPa, 60 s) under dark conditions at 25 °C. The transient transformation experiment was performed in triplicate. After infiltration, after 72 h of culture on MS medium, seedlings were subjected to GUS staining using the GUS Staining Kit (Coolaber, Beijing, China) according to the manufacturer’s protocol. Following 72 h of transient transformation, seedlings of *Chrysanthemum indicum* were subjected to 4 °C for 0 and 1 h. Leaf samples were sampled for DAB and NBT histochemical staining to detect ROS accumulation. Relative electrical conductivity (REC) was measured according to previously described methods [63]. The activities of catalase (CAT), superoxide dismutase (SOD), and peroxidase (POD), as well as malondialdehyde (MDA) content, were determined using the CAT Activity Assay Kit (Solarbio, Beijing, China), SOD Activity Assay Kit (Solarbio, Beijing, China), POD Activity Assay Kit (Solarbio, Beijing, China), and MDA Content Assay Kit (Solarbio, Beijing, China), respectively, following the manufacturer’s instructions.

### 4.10. Statistical Analysis

All experiments were performed with at least three independent biological replicates. Data are presented as mean ± standard deviation (SD). Statistical analyses were conducted using GraphPad Prism (v10.1.2). Comparisons between two groups were performed using Student’s *t*-test, while comparisons among multiple groups were assessed by one-way analysis of variance (ANOVA) followed by Dunnett’s multiple comparison test.

## 5. Conclusions

In this study, Genome-wide analysis of *Chrysanthemum indicum* has identified a total of 14 *CiHsf* genes. Analysis of gene structures, motifs, chromosomal localization, evolutionary relationships, and promoter elements revealed that the *CiHsf* gene family is structurally conserved and plays important roles in the growth and development of *Chrysanthemum indicum*. Expression analysis of *CiHsf* genes in different tissues and under low-temperature stress demonstrated that the *CiHsf* gene family is involved in developmental processes and cold stress response in *Chrysanthemum indicum*. Furthermore, transient overexpression of *CiHsf10* in *Chrysanthemum indicum* significantly reduced ROS accumulation under low-temperature conditions. This study provides a foundation for elucidating the roles of Hsf transcription factors in plant growth, development, and low-temperature stress response, and may facilitate related breeding efforts.

## Data Availability

RNA-seq data of low-temperature-treated *Chrysanthemum indicum* have been deposited in the NCBI Sequence Read Archive under the accession number PRJNA1391062.

## Supplementary Materials

The following supporting information can be downloaded at: https://www.mdpi.com/article/10.3390/plants15081149/s1, Table S1: List of all *Hsf* genes identified in the Chrysanthemum genome; Table S2: Conserved protein domain sequences of the *CiHsf* gene family; Table S3: The primer sequences used for qPCR; Table S4: The primer sequences used for *CiHsf10* PCR; Table S5: The full-length Hsf protein sequences of the three species; Table S6: Relative expression value; Figure S1: Electrophoretogram of *CiHsf10* gene cloning.
