# Supplementary material for: Genome-Wide Analysis of the Hsf Family and Functional Characterization of CiHsf10 Under Low-Temperature Stress in Chrysanthemum indicum
Source: Plants (Basel). 2026 Apr 9;15(8):1149. doi: 10.3390/plants15081149 (PMC13120352; doi:10.3390/plants15081149)
Supplement: Supplementary file 1 [file plants-15-01149-s001.zip › Supplementary Table_S1.pdf]

Table S1 List of all Hsf genes identified in the Chrysanthemum genome

Gene ID	Gene Name	Protein length (aa)	Molecular weight (kDa)	pI	Instability Index	GRAVY	Aliphatic Index	Subcellular localization prediction
CiHsf1	D.indicum_Contig615_G00009	326	37.05055	7.06	45.82	-0.671	65.80	nucleus
CiHsf2	D.indicum_Contig165_G00002	300	34.19195	4.76	56.12	-0.775	67.90	nucleus
CiHsf3	D.indicum_Contig27_G00089	355	40.69374	4.88	45.39	-0.603	77.13	nucleus
CiHsf4	D.indicum_Contig325_G00013	277	32.03734	5.51	53.62	-0.847	71.41	nucleus
CiHsf5	D.indicum_Contig118_G00059	285	31.76247	6.85	36.68	-0.831	60.49	nucleus
CiHsf6	D.indicum_Contig66_G00034	496	54.73662	9.04	33.00	-0.359	75.99	nucleus
CiHsf7	D.indicum_Contig166_G00148	323	37.61307	4.92	70.84	-0.793	69.97	nucleus
CiHsf8	D.indicum_Contig166_G00149	334	39.02066	5.14	70.43	-0.836	69.13	nucleus
CiHsf9	D.indicum_Contig375_G00003	325	37.72305	4.88	67.52	-0.802	69.85	nucleus
CiHsf10	D.indicum_Contig257_G00037	357	40.36809	4.86	55.78	-0.684	71.48	nucleus
CiHsf11	D.indicum_Contig1196_G00006	447	50.05834	4.99	49.36	-0.738	64.77	nucleus
CiHsf12	D.indicum_Contig779_G00011	283	32.74598	4.99	50.44	-0.682	68.45	nucleus
CiHsf13	D.indicum_Contig31_G00250	234	27.37070	4.72	73.30	-0.774	82.48	nucleus
CiHsf14	D.indicum_Contig28_G00315	249	28.14777	5.39	43.25	-0.581	66.55	nucleus

Addition Table S1 List of primers used in this study

Primer Name	Primer Sequence(5' -3')
EF1α-qF	TTTTGGTATCTGGTCCTGGAG
EF1α-qR	CCATTCAAGCGACAGACTCA
CiHsf1-qF	TGTCGGAAGACAACGAACGG
CiHsf1-qR	GCAACGGGTTTCACATGGTT
CiHsf2-qF	GGCCCTCGGATTTTCAGACA
CiHsf2-qR	ATACTGAGACAGCTCGCCAC
CiHsf3-qF	ATCAGCCACCAGTAGAATCACC
CiHsf3-qR	GAATCCATGCCCTCATCCACT
CiHsf4-qF	GACTATGGCGACGGGTCAAC
CiHsf4-qR	GCTCTTCCAAATCCTTGCATTTCT
CiHsf5-qF	ACAGTGTGACGATCTCGTGG
CiHsf5-qR	CGAGTTCACATCACCCGTCA
CiHsf6-qF	ATGCTGGTGAAGGATGTGGT
CiHsf6-qR	TACTGGAAACCGGGCGTAAC
CiHsf7-qF	CCCTTTCATTACAGCCCCGT
CiHsf7-qR	ATCGGGTGGTTCCTCAGTTG
CiHsf8-qF	TGTCTGGGATTTGTGTCGGT
CiHsf8-qR	TCCCATCTGTCTGGGTCCAC
CiHsf9-qF	AGGCTGAGACAACTGCAACA
CiHsf9-qR	GAACTCGGGGTTGCTAAACG
CiHsf10-qF	ACCCCACGAACCAAATCCTG
CiHsf10-qR	TCAATCCCACCACCAAACGG
CiHsf11-qF	GATGGCGGGAGTCCACAAAT
CiHsf11-qR	TGTGAACGACGTGCCAGTAT
CiHsf12-qF	TCAGCCACCGTTTGTAGAGC
CiHsf12-qR	CTCTCACTGGTGAACCACACA
CiHsf13-qF	CGACCCATTCATTACAGCCC
CiHsf13-qR	ATCAGGTGGTTCCTCAGTCG
CiHsf14-qF	CGATGAAGGGGGTTGAAGGT
CiHsf14-qR	TCGACAACGTCATCCTCCAAA
