# Supplementary material for: Genome-Wide Analysis of the Hsf Family and Functional Characterization of CiHsf10 Under Low-Temperature Stress in Chrysanthemum indicum
Source: Plants (Basel). 2026 Apr 9;15(8):1149. doi: 10.3390/plants15081149 (PMC13120352; doi:10.3390/plants15081149)
Supplement: Supplementary file 1 [file plants-15-01149-s001.zip › Supplementary Table_S2.pdf]

Table S2 Conserved protein domain sequences of the *CiHsf* gene family.

Gene ID	Subgroup	DBD	HR-A/B	NLS	NES	AHA motif
CiHsf1	B4	22-114	191-226	(293)KKRLHPEYGSNATMVEAHKARLV	nd	nd
CiHsf2	A3	8-77	94-136	(159)PRFKKGKGL	(178-192)IPVDDTVRNFDHVPF	(279-291)WNLEAASGAENIL
CiHsf3	A8	10-103	138-191	(214)PSKTIIKPVTDDIDTIVAPHDRAIVKYQPP	(248-262)DSLEMGLTLEEIEDI	(320-335)WSSEMETDEYRFLTEG
CiHsf4	A6	34-85	113-163	(193)IMNKRRRFID	(216-230)NPSGFEMSELDELAL	(262-270)EFWEELFNE
CiHsf5	B1	6-99	151-189	(244)EDKKKRGRDEINNSLGVQRKEMKMNNCNNATP	nd	nd
CiHsf6	B4	106-162	253-270	(367)LSKKRMHPEY	nd	nd
CiHsf7	A2	32-125	149-202	(233)EIGRKRRLTMT	(270-284)ESHDNLWEELLNVDL	(303-312)DWYEDLQELV
CiHsf8	A2	33-126	154-203	(234)EIGRKRRLTMT	(271-285)DSHDNLWEELLNVDL	(305-313)WDEDLQELV
CiHsf9	A2	34-127	152-204	(235)EIGRKRRLTMT	(272-286)DSHDNLWEELLNVDL	(306-314)WDEDLQELV
CiHsf10	A3	54-147	163-214	(274)FFKGKNVLEMEPEYLLEPFGGGIDDISVKQED	(280-294)PEYLLEPFGGGIDDI	(302-313)WSELGNYELPEL
CiHsf11	A5	17-110	128-183	(214)YNKKRRLPSD	(329-343)PCEEAEGNLSCLLNL	(405-414)DVFWEQFLTE
CiHsf12	A8	1-83	113-166	nd	(229-243)TSDEVKDLLENMDVM	(255-264)FVFHDMSDSD
CiHsf13	A2	2-40	64-114	(144)EIGRKRRLTMT	(181-195)ESHDNLLEELLNVDL	(215-223)WDEDLQELV
CiHsf14	B2	5-98	144-183	(228)VSLGTKRARTSLE	nd	nd

Notes: nd, not detected.
