# Supplementary figures and images for: Genome-Wide Analysis of the Hsf Family and Functional Characterization of CiHsf10 Under Low-Temperature Stress in Chrysanthemum indicum
Source: Plants (Basel). 2026 Apr 9;15(8):1149. doi: 10.3390/plants15081149 (PMC13120352; doi:10.3390/plants15081149)

**Figure S1.** Electrophoretogram of *CiHsf10* gene cloning.

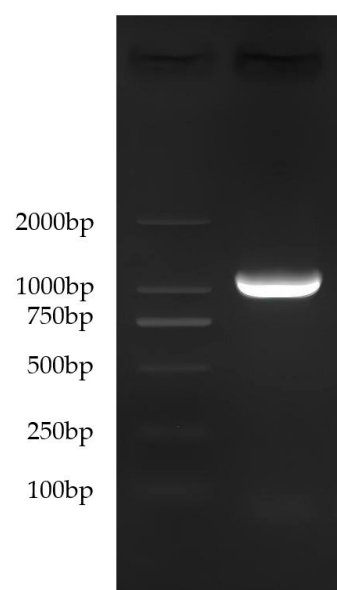

Supplement: Supplementary file 1 [file plants-15-01149-s001.zip › Supplementary Figure_S1.pdf]
